# Bilingualism and Biliteracy in Down Syndrome: Insights From a Case Study

**DOI:** 10.1111/lang.12179

**Published:** 2016-06-01

**Authors:** Kelly Burgoyne, Fiona J. Duff, Dea Nielsen, Anastasia Ulicheva, Margaret J. Snowling

**Affiliations:** ^1^ University College London; ^2^ University of Oxford; ^3^ University of Sheffield; ^4^ University of Hong Kong

**Keywords:** Down syndrome, bilingualism, biliteracy, case study

## Abstract

We present the case study of MB—a bilingual child with Down syndrome (DS) who speaks Russian (first language [L1]) and English (second language [L2]) and has learned to read in two different alphabets with different symbol systems. We demonstrate that, in terms of oral language, MB is as proficient in Russian as English, with a mild advantage for reading in English, her language of formal instruction. MB's L1 abilities were compared with those of 11 Russian‐speaking typically developing monolinguals and her L2 abilities to those of 15 English‐speaking typically developing monolinguals and six monolingual English‐speaking children with DS; each group achieving the same level of word reading ability as MB. We conclude that learning two languages in the presence of a learning difficulty need have no detrimental effect on either a child's language or literacy development.

## Introduction

As the number and visibility of children learning multiple languages in childhood has increased, so too has research interest in the impact of bilingualism on the development of children's linguistic, cognitive, and literacy skills. Bilingual children form a heterogeneous group, and their experiences in terms of how and when they learn their languages, as well as whether they develop literacy skills in a language, are diverse. While some children learn to read and write in their dominant language, others may begin literacy instruction in a language they are still acquiring, while still others may be learning to read in all of their languages (Bialystok, Luk, & Kwan, 2005). As such, quantifying the impact of bilingualism on literacy development is a complex process.

## Bilingualism and Literacy Development

One important consideration is the influence of bilingualism on the cognitive processes that underpin literacy. Research from both monolingual and bilingual children has highlighted the importance of oral language, phonological awareness, and letter‐knowledge for reading development (August & Shanahan, 2006). Oral language skills, particularly vocabulary, are consistently found to be more limited in bilingual children's individual languages than in monolingual children, and these weaknesses are related to limitations in reading comprehension (Babayigit, 2015; Burgoyne, Whiteley, & Hutchinson, 2011; Cobo‐Lewis, Pearson, Eilers, & Umbel, 2002; Melby‐Lervåg & Lervåg, 2014). However, both phonological awareness (PA) and letter‐sound knowledge have been found to develop very similarly in monolingual and bilingual children, and in certain instances bilingual children show superior PA abilities (August & Shanahan, 2006; Bialystok et al., 2005; Bialystok, Majumder, & Martin, 2003; Chiappe, Siegel, & Gottardo, 2002). Furthermore, there is now evidence to suggest that having sufficient knowledge of multiple languages is related to advantages in creativity, cognitive flexibility, attention control, working memory, and broader metalinguistic awareness (Adesope, Lavin, Thompson, & Ungerleider, 2010; Reyes, 2012). Thus, the impact of bilingualism on cognitive development is not uniform and is related to positive, negative, or neutral effects depending on the specific skill being considered.

An additional feature of bilingual children's literacy development is the potential impact and transfer of skills from one language to the other. A prominent idea within the area of crosslinguistic transfer contends that children's language skills are underpinned by a central processing system, and it follows that children's abilities in one language should be related to their abilities in the other (Cummins, 1991). Where such transfer effects do exist, they may allow bilingual children to take advantage of skills in one language to support development and learning of literacy skills in their other language. This idea has been central to research that considers which cognitive and linguistic skills are correlated across languages and thereby believed to show transfer.

Research into the literacy skills of bilingual children has tended to focus on literacy outcomes for children in their second language (L2), and research on biliteracy—learning to read in two language—is still sparse (August & Shanahan, 2006; Reyes, 2012). Bialystok (1997) found that 4‐ to 5‐year‐old bilingual French‐English and Chinese‐English children showed more advanced understanding of symbolic representation as compared to monolingual children, which was interpreted as a result of exposure to multiple languages and its support of children's metalinguistic understanding. In a study of children in their first year of literacy instruction, Bialystok et al. (2005) examined the phonological awareness and literacy skills of Cantonese‐English bilingual, Hebrew‐English bilingual, Spanish‐English bilingual, and monolingual English children. These specific language pairs allowed for the comparison of effects on literacy when children were learning to read in two alphabetic scripts with the same alphabet (Spanish‐English bilinguals), two alphabetic scripts with different alphabets (Hebrew‐English), and two languages that used different writing systems. Results suggested that bilingual children learning two alphabetic scripts showed advantages for both phonological awareness and decoding, but these benefits were more limited in Cantonese‐English bilingual children, suggesting that the specific characteristics of the language pairs to be learned will influence the extent of cross‐language transfer and facilitation. Other research has also suggested that writing systems may place different cognitive demands on readers; therefore, the attributes of children's individual languages are an important consideration in terms of how biliteracy affects cognition (Wang, Perfetti, & Lui, 2005).

## Bilingual Literacy and Down Syndrome (DS)

With these findings as a backdrop, we note that most research has been undertaken on bilingualism in typically developing children. However, a concern continually expressed by teachers and clinicians is whether or not to encourage bilingualism in a child with language or learning difficulties and, even more so, encourage biliteracy. There is currently a dearth of information to inform such clinical decisions. Here, we present the case of MB, a girl with DS who is bilingual and biliterate in Russian (her first language [L1]) and English (L2). There are very few studies of bilingualism in DS, and our description of MB provides the first detailed study of biliteracy development in such a child. We believe that this case is relevant to understanding bilingualism and reading development in atypical populations and has practical implications for parents and practitioners working with multilingual children with intellectual disability.

DS is a chromosomal disorder caused by trisomy of chromosome 21 (Wiseman, Alford, Tybulewicz, & Fisher, 2009) and is one of the most common causes of learning disability. DS is associated with a particular profile of cognitive strengths and weakness, though there is considerable variability in the phenotype. Language development is significantly impaired in DS, with broad deficits across language domains relative to nonverbal ability; grammar is a particular area of difficulty, and verbal short‐term memory is particularly impaired (Naess, Melby‐Lervåg, Hulme, & Lyster, 2012). Despite these language difficulties, many children with DS can learn to read. Attainment levels vary widely with some children reading at levels commensurate with chronological age (e.g., Burgoyne et al., 2012; Hulme et al., 2012). Performance on reading and related tasks is, however, typically uneven: Although word reading skills are commonly better than expected given levels of phonological awareness (Lemons & Fuchs, 2010) and nonword reading (Hulme et al., 2012; Naess et al., 2012), reading comprehension is typically weak and commensurate with oral language (Groen, Laws, Nation, & Bishop, 2006; Nash & Heath, 2011).

The predictors of individual differences in reading among children with DS appear to be different from those in typically developing children. When typically developing children are learning to read in an alphabetic system (such as English and Russian), word‐level reading skills are predicted by letter knowledge and phoneme awareness, while broader oral language skills (e.g., vocabulary and grammar) predict reading comprehension (e.g., Hulme et al., 2012). However, phoneme awareness is a particular area of difficulty for many children with DS, and there is evidence that vocabulary and grammar, not phoneme awareness, typically predict growth in word reading (Burgoyne et al., 2012; Hulme et al., 2012; Steele, Scerif, Cornish, & Karmiloff‐Smith, 2013). By contrast, the predictors of reading comprehension are similar in children with and without DS; problems in understanding what is read are related to weaknesses in language and verbal memory (Levorato, Roch, & Beltrame, 2009; Nash & Heath, 2011) in DS, just as they are in typical development.

Given the learning disability associated with DS, it is reasonable to hypothesize that exposure to more than one language may pose particular problems for language learning. However, a small number of studies suggest that this is not the case. The first published reports of bilingualism in individuals with DS came from case studies (Vallar & Papagno, 1993; Woll & Grove, 1996). Vallar and Papagno reported the case of a trilingual 23‐year‐old woman (FF). FF was born to Italian‐speaking parents (her father was also fluent in English) and was schooled in Italy. In addition to Italian, she learned English as a child (to a level at which she could converse and understand) and French (at which she was less proficient). Examination of FF's cognitive abilities in her L1 demonstrated good acquisition of language and vocabulary, despite widespread cognitive impairment. Unlike the typical DS profile, FF showed normal phonological short‐term memory skills, and the authors postulated that this might have been why she had acquired vocabulary well. Though reading skills were not a primary focus of the study, the authors reported that FF showed virtually errorless, albeit slow, reading (in L1) and that she performed well in discriminating initial sounds in words (though FF was unable to parse phonemes). Therefore, exposure to more than one language did not, in this case, restrict L1 development; however, given she was studied by the researchers in her adulthood, it is difficult to know about the course of language acquisition.

Kay‐Raining Bird and colleagues reported the language profile of eight bilingual children with DS aged 6 years 5 months with an average mental age of 2 years 7 months (Kay‐Raining Bird et al., 2005). All children had English as their L1 and were either English‐dominant or balanced bilinguals; the majority had French as their L2. They had received intensive ongoing and prolonged input in both languages. Their performance was compared to that of 14 monolingual children with DS of the same chronological age and two younger, typically developing groups of children of the same mental age: 18 monolingual children (*M*_age_ = 2;07) and 11 bilingual children (*M*_age_ = 2;10). All children were recruited in the early stages of language development, with a minimum expressive vocabulary of 100 words. As expected, both DS groups showed the classic profile of lower verbal than nonverbal skills. Although the DS bilingual group did not differ from typically developing bilingual controls in receptive or expressive vocabulary, their mean length of utterance was shorter, and qualitative analyses revealed expressive language difficulties (Feltmate & Kay‐Raining Bird, 2008). However, there was no difference between the monolingual and bilingual groups with DS on any of the L1 measures, suggesting that bilingualism in DS does not have a detrimental effect on L1 language competence. Consistent with this finding, Cleave, Kay‐Raining Bird, Trudeau, and Sutton (2014) showed that monolingual and bilingual children with DS performed equally well on a fast‐mapping task that required the pairing of novel phonological representations (new names) to semantic referents, but the researchers did not test the children in their L2.

Building on these findings, Edgin, Kumar, Spano, and Nadel (2011) examined the specific cognitive effects of L2 exposure on wider cognitive function in DS. Thirteen individuals with DS (aged 7–18 years) who had frequent exposure to a language other than English (predominantly Spanish) were compared to 28 monolinguals matched for age, gender, IQ, and socioeconomic status. Exposure to the additional language ranged between 1 and 11 hours per day, predominantly from family members who spoke another language at home. No differences between monolingual and L2 exposed groups were found on measures of English language skill or on tests of memory and executive function.

## The Current Study

Taken together, these studies suggest that L2 exposure does not affect language or wider cognitive outcomes for individuals with DS. However, there are a number of limitations to this work. Language skills are not always objectively measured or clearly reported in both languages, making it difficult to assess the extent to which individuals are bilingual. Sampling issues also pose potential problems for interpreting this work: A wide age range (Edgin et al., 2011) may mask cognitive advantage or disadvantage at particular ages or stages in development (Goral & Conner, 2013) or when comparison groups (matched for mental age) are significantly younger and therefore have less language exposure (Kay‐Raining Bird et al., 2005). Furthermore, the reading abilities of bilingual individuals with DS are rarely reported. To our knowledge, only one study has looked at reading in more than one language in an individual with DS (Nelson, Damico, & Smith, 2008). The study reported eye movements during text reading in Spanish (L1) and in English (L2), which was the language of schooling from age 4, with no differences being observed between the pattern in each language. Conclusions are, however, limited because this was a single‐case study, and the authors did not measure the cognitive ability, word‐level reading, or language skills underpinning literacy.

Thus, there is a lack of information about the impact of bilingualism on reading development in DS. Because the cognitive and linguistic profile of children with DS does not appear to be different in bilingual and monolingual cases, it might be considered reasonable to speculate that the same will be true for reading. However, an alternative prediction is possible. As discussed above, bilingual advantages have been reported in both metalinguistic and metacognitive skills (Adesope et al., 2010; Bialystok, 1999; Bialystok & Martin, 2004). These include benefits to phonological processing and particularly to phonological awareness, which are highly relevant to learning to read (Bialystok et al., 2005).

If bilingualism confers advantages in phonological awareness, this advantage may be particularly helpful for individuals with DS, for whom phonological awareness is typically weak. In this case, we might expect bilingualism to have a positive effect on word‐level reading in both L1 and L2, while reading comprehension (in L1 and L2) should be constrained by the level of oral language competence specific to the writing system in which the child is reading. Alternatively, the potential demands of learning more than one language on already limited cognitive resources may have negative consequences for reading development in DS.

The current case study is of particular interest as MB reads both English, which uses the 26 letters of the Latin alphabet to represent 46 phonemes, and Russian, in which the Cyrillic alphabet uses 33 letters with which to represent 43 phonemes. English is a deep orthography with many inconsistencies in letter‐sound correspondences (Seymour, Aro, & Erskine, 2003; Ziegler et al., 2010), and Russian is a transparent orthography which embodies mostly regular and consistent mappings between orthography and phonology (Ulicheva, Coltheart, Saunders, & Perry, 2016), although stress assignment in Russian is highly irregular (Jouravlev & Lupker, 2014). Thus, MB faces the challenge of mastering two symbol systems and, across languages, inconsistencies in the mapping of written symbols onto their pronunciations (e.g., letter “p” is typically read as /p/ in English but /r/ in Russian). Given the language learning difficulties associated with DS, these inconsistencies might reasonably be considered to pose a challenge.

The main aim of the current study was therefore to examine biliteracy in DS through a case study and to investigate the extent to which learning two languages might affect the cognitive profile of such an individual as well as the possible impact on literacy development in each language. We first considered MB's general cognitive and linguistic abilities before turning to examine her reading and related skills in English (L2), the language in which she is receiving formal instruction, and in Russian (L1). Specifically, we addressed the following questions:

1. What is MB's cognitive profile? To address this, we compared her performance on nonverbal and verbal tasks. We also assessed her proficiency in L1 and L2 to judge whether she is a balanced bilingual.
2. What are MB's L2 reading skills and her reading progress? To address this, we monitored MB's L2 reading progress over time.
3. To what extent does bilingualism confer an advantage (or disadvantage) for L2 reading? We compared MB's reading ability to that of two comparison groups matched for word reading: (a) Comparison with a group of monolingual English‐speaking typically developing children allowed us to consider whether MB's broader reading skills show the typical pattern for her word reading level or whether, consistent with the typical DS profile, she shows better word reading than nonword reading, phonological awareness, and reading comprehension; (b) Comparison with monolingual English‐speaking children with DS allowed us to consider potential effects of bilingualism on phonological awareness and nonword reading skills, relative to peers with DS.
4. Does MB show the same reading profile in L1 (Russian) as in L2 (English)? Relatedly, does she perform better in English (the language of formal literacy instruction) or Russian (given the relatively high consistency and regularity of orthography‐to‐phonology correspondences in this orthography)? To address this question, we compared MB's reading abilities in English and Russian and compared her L1 reading abilities with those of monolingual Russian‐speaking typically developing children.

## Method

### Participants

MB is a child with DS who is functioning in the moderate to severe range on cognitive tests, with better verbal than nonverbal abilities. Indeed, considering her diagnosis, MB's language skills are well developed. MB was born in Belarus to multilingual parents (Russian‐English‐Belarusian). The family moved to the United Kingdom when MB was 6 months old. She is a sequential bilingual learner, having acquired Russian as her L1 at home with limited exposure to English until school entry (at age 4). MB's parents read to her in Russian daily and extensively and began teaching her to read words in Russian (using a whole‐word strategy) at the age of 30 months. They judged her to be “at ceiling” on Russian sight word reading at school entry. At this point, MB could also read around 20 words in English (also due to instruction at home). All of her formal education had been delivered in English. Background information regarding MB's health, and her language and literacy development, were obtained through a semi‐structured interview with her parents. Relevant information is summarized in Table 1.

**Table 1:** Parental report regarding MB's health and language and literacy development

Health background	
Hearing	Minor binaural hearing loss
Vision	Near‐sighted; nystagmus; corrected with glasses
Engagement with support services (English provision)	
Portage	Weekly from 14 months to 3 years; fortnightly thereafter until 5 years
Speech and language	Bimonthly from 3 to 5 years
therapy	
Literacy	At (mainstream) school entry until time of testing: comprehensive one‐to‐one English literacy support from a teaching assistant, including a 40‐week language and literacy intervention (Burgoyne et al., 2012)
Parental background	
Father	Trilingual (Russian, Belarusian, English); holds a PhD
Mother	Bilingual (Russian, English); holds a PhD
Home environment	
Language	Almost exclusively Russian; English language exposure limited until school entry
Literacy	Almost exclusively Russian; parents have been reading to MB daily and extensively in Russian since before school; approximately 75 Russian and 50 English story books at home
Developmental milestones	
Language	In Russian: first spoken words at 24 months; approximately 50 words by 3.5 years; spoken vocabulary size judged to be “beyond estimation” by parents at time of testing
	In English: approximately 10 spoken words at 4 years and 50 at 5 years; spoken vocabulary judged to be “beyond estimation” at time of testing, but smaller than in Russian
Literacy	In Russian: began learning to read sight words at 30 months; judged to be “at ceiling” at school entry
	In English: approximately 20 sight words at school entry

MB's first assessment point for the current study was immediately following a 40‐week language and literacy intervention (see Burgoyne et al., 2012), when she was 6 years 11 months (T1). MB was subsequently assessed at age 7 years 9 months (T2), when her performance was compared to that of three monolingual comparison groups matched on word reading ability (described below). MB was assessed again in English at age 9 years 6 months to monitor her progress (T3).

There are several issues to consider when comparing children with DS to typically developing comparison groups regarding the choice of matching variable. Matching on nonverbal mental age or language ability results in a comparison group that is much younger in age (e.g., Kay‐Raining Bird et al., 2005). The utility of this approach is severely limited and not easily applicable to studies of reading, because the comparison group would have significantly less (or perhaps no) exposure to literacy instruction. By matching the monolingual comparison groups to MB on word reading ability, we are able to examine whether MB shows a similar profile across her reading and language skills, or whether her experience of more than one language has led to a different pattern of strengths and weaknesses.

#### DS Monolingual English Comparison Group

This group comprised six English‐speaking children with DS who had completed the same intervention (Burgoyne et al., 2012) as MB (*M*_age_ = 9;05, *range* = 8;01–10;06). They were matched to MB's word reading ability at T1 using the Early Word Reading (EWR) test from the York Assessment of Reading for Comprehension (YARC).

#### Typically Developing Monolingual English Comparison Group (TDE)

Fifteen children from the same school as MB acted as English‐speaking monolingual controls. They were a similar age to MB at T2 (*M*_age_ = 7;07, *range* = 7;02–7;10) and were matched in word reading ability at T2, using the EWR test.

#### Typically Developing Monolingual Russian Comparison Group (TDR)

Eleven children living in Moscow acted as Russian‐speaking monolingual controls. They were matched to MB's L1 reading ability at T2 using a Russian word reading test (described below); these children were between 9 and 21 months younger than MB at this test point (*M*_age_ = 6;05, *range* = 6;00–7;00).

### Design

We designed the assessment battery to assess the cognitive and linguistic skills that underpin MB's literacy in her L1 (Russian) and L2 (English). We employed a range of standardized (English‐only) and bespoke (Russian‐English) tests to assess general cognitive ability, vocabulary, and literacy skills. MB was tested at three time points (T1, T2, T3) and at T2 completed tests in both English and Russian. Children in comparison groups were tested at T2 only and completed tests in their spoken language (English or Russian). Appendix S1 in the Supporting Information online provides an overview of the tests completed by each group of participants.

### Tests of General Cognitive Ability

To estimate MB's level of general cognitive ability, which could then be compared to her language and literacy skills, we administered five standardized tests. We used these tasks to consider whether MB's cognitive profile was typical of DS with higher nonverbal than verbal abilities.

#### Nonverbal Skills

These skills were assessed using two subtests from the Wechsler Preschool and Primary Scale of Intelligence (WPPSI‐III; Wechsler, 2003). For Block Design, children were required to manipulate blocks to copy designs of increasing complexity. For Object Assembly, they arranged jigsaw puzzle pieces of increasing numbers in order to complete pictures. In addition, visual‐spatial memory was assessed using Block Recall (Working Memory Test Battery for Children [WMTBC]; Pickering & Gathercole, 2001). The experimenter tapped blocks in sequences that increased in length, and children were asked to copy the sequences exactly.

#### Verbal Memory

The Digit Recall and Word Recall subtests of the WMBTC were administered in English to assess verbal short‐term memory. Children were required to repeat increasingly longer lists of digits and of words, respectively.

### Measures of Language Skills

To provide an assessment of MB's oral language skills, we used measures of receptive and expressive vocabulary. We chose to focus on vocabulary measures because these are known predictors of reading in children with DS. Two tests assessed MB's English vocabulary. The British Picture Vocabulary Scale (BPVS‐III; Dunn, Dunn, Styles, & Sewell, 2009) assesses receptive vocabulary. Target words are presented verbally and the child is required to point to the corresponding picture from four options. The Expressive Vocabulary subtest from the Clinical Evaluation of Language Fundamentals (CELF‐IV; Semel, Wiig, & Secord, 2003) requires picture naming.

In order to compare MB's English language skills with her Russian language ability, the English test items from the BPVS‐III and the CELF‐IV were translated into Russian independently by two native speakers of Russian, with all translated targets and distractors checked to ensure they were culturally, linguistically, and age appropriate in Russian. Items were discounted in both languages if translation was considered inappropriate or (in the receptive measure) if the target word in Russian was not deemed distinct from the distractors. Only items that both native speakers considered unambiguous were included in the final assessment.

### Measures of Literacy Skills

In order to enable crosslinguistic comparisons, bespoke literacy tasks with parallel Russian‐English versions were created. All reading and phonological measures were adapted from those of Schwartz (2006). The Russian tests were used as the benchmark, and English stimuli were selected to be of equivalent difficulty. Every effort was made to maximize matching of stimuli across languages in terms of word class, number of syllables and phonemes, consonant‐vowel structure, and word frequency, according to English norms from the Children's Printed Word Database (Masterson, Stuart, Dixon, & Lovejoy, 2003). Parallel test items for each of the bespoke literacy tasks can be found in Appendixes S2 to S5 in the Supporting Information online.

#### Phonological Awareness (T2)

Phonological awareness was assessed using bespoke measures of syllable and phoneme deletion and phoneme isolation. Each correct response received a score of 1 point, giving a total score out of 10 for each test in each language. Syllable deletion: Children were asked to delete five initial and five final syllables from two‐syllable words. For each item, children heard the word, repeated its full form, and said the word again without the target syllable. Items were such that a meaningless syllable was deleted from a word to create a new meaningful word in Russian; but in English, a meaningful syllable was deleted to leave a meaningful target word. Phoneme deletion: This task required deletion of four initial, four final, and two medial phonemes from one‐ to two‐syllable words. For each item, children heard the word, repeated its full form, and said the word again without the target phoneme. The correct answer was a new meaningful word for all items in English but for only half the items in Russian. Phoneme isolation: Children were required to isolate 10 initial and 10 final phonemes from words ranging in length from one to three syllables. For each item, children saw a picture, heard the corresponding word, and were asked to say the sound which was at the beginning or end.

#### Letter Knowledge (T2)

The Letter Knowledge subtest from the YARC was used to measure the ability to provide the sounds for 26 graphemes and six digraphs from English orthography. To test Russian letter knowledge, children were asked to provide the sounds for all 33 letters of the Russian alphabet. A score of 1 was given for each correct letter sound. When comparing MB's scores across English and Russian, a percentage score was derived representing correct responses to single graphemes (26 in English, 33 in Russian).

#### Word and Nonword Reading

The EWR test and the Single Word Reading (SWR) test from the York Assessment of Reading for Comprehension (YARC) provided assessments of English word reading. Both tests require children to read aloud lists of words that gradually increased in difficulty. The Graded Nonword Reading Test (Snowling, Stothard, & McLean, 1996) was administered to English‐speaking participants. In this test, children were presented with 20 nonwords to read aloud that increased in difficulty (e.g., hast, sloskon). A score of 1 was given for each nonword read correctly.

Word and nonword reading was also assessed using bespoke tests, which used words and nonwords matched for number of phonemes and, in most cases, consonant‐vowel structure in English and Russian. On both tests, children were asked to read aloud a list of 15 words/nonwords as quickly and accurately as possible. Items on the English and Russian tests ranged from one to four syllables. Each correctly read word/nonword received a score of 1 point.

#### Reading Comprehension

English‐speaking participants were administered the Beginner passage and Level 1 and 2 passages from the YARC Passage Reading test (Hulme et al., 2011) at T2; MB completed this test again at T3. Children were timed while they read each passage aloud. After each passage, they were asked eight related questions. Raw scores for prose reading accuracy (errors) and comprehension were computed by totaling across all three passages. For MB, standard scores were based on Level 1 and 2 passages at T2, and Level 2 and 3 passages at T3.

### Procedure

Informed parental consent was given for children to take part. Children were tested individually by researchers or students trained in test administration. All children in the comparison groups were assessed at school; the TDE and DS groups in two sessions and the TDR group in one session. MB was assessed in English in school at T1. At T2, MB was visited at home on three occasions. In sessions 1 and 2, spaced 2 weeks apart, MB completed the matched tests and standardized tests in English. Session 3 occurred 3 weeks later, when she completed the Russian tests. MB's T3 assessment was also completed at home.

### Statistical Methods

We followed the procedure of Groen et al. (2006) for statistical analysis. We employed independent *t* tests that were modified for small sample sizes, using the program SINGLIMS.EXE (Crawford & Garthwaite, 2002). For any given measure, a *t* test compared MB's score with the mean of a comparison group. The procedure supplied “point” estimates of the percentage of the population that would have been expected to score lower than MB, with 95% confidence intervals, where the population was reflected by the comparison group. Crawford and Garthwaite suggest that a point estimate of less than 2.5% represents a clear deficit in performance; therefore, an estimate that is equal to or larger than 97.5% might be taken to represent a clear advantage. In all cases, two‐tailed tests were used.

## Results

We first considered MB's performance on tests of general cognitive abilities and language to ascertain her cognitive profile and to determine the extent to which she can be considered a balanced bilingual. We then analyzed data for her literacy skills in Russian (L1) and in English (L2), the language in which she was receiving formal instruction.

### What Is MB's Cognitive Profile?

As would be expected for a child with DS, MB's general cognitive abilities were significantly below those of the TDE group (Table 2); she gained a scaled score of 1 on Block Design and Object Assembly (IQ equivalents = 55) and scored below the first centile for Block Recall (IQ equivalent < 55). On tests of verbal memory, she gained standard scores of 73 (Digit Recall) and 66 (Word Recall).

**Table 2:** English performance on tests of language, verbal memory, and nonverbal ability for MB (score) at T2, compared with that of typically developing monolingual English children and children with Down Syndrome (DS; means, standard deviations), matched to MB on word reading accuracy

Measure (max score)	MB	TDE (*n* = 15)	Point estimate^1^ (95% CI)	DS (*n* = 6)	Point estimate^2^ (95% CI)
Nonverbal ability					
Object assembly (37)	12	30.73 (5.79)**	0.37 (0.00–2.63)	15.67 (8.04)	34.51 (9.97–65.88)
Block design (40)	20	28.40 (3.48)*	1.74 (0.03–8.31)	20.67 (3.27)	42.85 (15.75–73.05)
Block recall (44)	7	22.40 (3.87)**	0.09 (0.00–0.76)	15.33 (4.23)	6.39 (0.04–30.23)
Vocabulary					
Receptive (135)	40	92.27 (11.78)**	0.04 (0.00–0.32)	62.00 (12.45)	8.00 (0.10–33.75)
Expressive (54)	16	35.40 (6.49)*	0.59 (0.00–3.81)	17.17 (4.92)	41.72 (14.93–72.11)
Verbal memory					
Digit recall (54)	20	22.60 (2.47)	16.27 (4.67–34.30)	16.33 (3.83)	79.22 (47.70–97.23)
Word recall (42)	12	16.80 (2.86)	6.32 (0.69–19.23)	12.00 (1.67)	50.00 (21.18–78.82)

Notes. ^1^Percentage of the English typically developing population aged 7 years estimated to perform below MB's score

^2^Percentage of the English DS population aged 8–10 years with age‐appropriate word reading estimated to perform below MB's score. CI = confidence interval.

^*^ *p* < .05, ^**^ *p* < .01, ^***^ *p* < .001.

Turning to language skills, Table 2 (center columns) shows that MB was performing significantly below the TDE comparison group on measures of vocabulary, particularly receptive vocabulary. When adjusting MB's vocabulary scores for additional words known in Russian, her receptive vocabulary remained significantly below that of her age‐peers (raw score changed from 40 to 52, point estimate from .04% to .26%). When making similar adjustments for her expressive vocabulary (raw score increased from 16 to 21), the difference was marginally statistically significant (*p* = .05; point estimate increased from .59% to 2.48%). By contrast, MB performed similarly to the comparison group on verbal short‐term memory tasks.

There were no significant differences between MB and the monolingual children with DS (Table 2, rightmost columns). While MB's receptive vocabulary in English did appear weaker, adjustment for the number of items known in Russian (but not English) took her raw score from 40 to 52, and the point estimate increased from 8.00% to 24.53%, bringing her more in line with the monolingual DS children. When similar adjustments were made for MB's expressive vocabulary, the raw score increased from 16 to 21, and the point estimate from 41.72% to 74.83%. Her nonverbal abilities were also in line with those of her peers with DS. MB's test scores on the matched Russian and English language tests are reported in Table 3. MB is performing at a similar level in both languages, which suggests that MB can be considered a balanced bilingual.

**Table 3:** Comparison of MB's performance on matched Russian (testing age = 7;10) and English (testing age = 7;09) tasks

Measure (max score)	Russian score	English score
Receptive vocabulary (84)	44	40
Expressive vocabulary (54)	13	16

### What Are MB's L2 Reading Skills and to What Extent Does Bilingualism Affect Reading Development?

For an initial evaluation of MB's English reading skills, we considered her age‐standardized reading scores over a 2.5‐year period (see Table 4). MB's reading accuracy and fluency scores consistently fell in the average range, that is, at the same level as her typically developing peers, even though English is her L2. However, as expected for a child with DS, her reading comprehension scores fell in the low‐ to below‐average range. To benchmark her reading ability against that typically observed among children with DS, we used data from an opportunity sample of 51 children with DS (Burgoyne et al., 2012), assessed at T1 in the present study. This sample (*M*_age_ = 8;08, *range* = 6;11–11;09) gained an average raw score on the EWR (max = 30) of 13.41 (SD = 10.47). Despite being the youngest child in the sample (6;11 at that time), MB obtained a raw score of 28 on this same test (equivalent to a standard score of 105). While 91.3% (95% CI = 84.21–96.22) of the DS population represented by this sample were estimated to gain lower scores than MB, the difference between her score and that of the sample mean was not significant (*t* = 1.38, *p* = .17).

**Table 4:** MB's standardized reading scores in English across a 2.5‐year period

Age at	Early word	Single word	Prose reading	Prose reading	Prose reading
testing	reading	reading	accuracy	rate	comprehension
6;11	105	98	—	—	—
7;09	95	87	93	95	86
9;06	—	91	92	89	79

Notes. Normative values: *M =* 100, *SD* = 15, *M*_range_ = 85–115.

### Does Bilingualism Confer an Advantage (or Disadvantage) for L2 Reading?

MB's scores on tests of phonological awareness and letter‐sound knowledge are shown in the upper rows of Table 5. Although MB could read words as well as the TDE group, and her knowledge of letter sounds was similar, her phonological awareness was less well developed; she scored significantly less well than the TDE group in syllable deletion, and marginally so in phoneme deletion and in phoneme isolation of initial sounds. Her performance on phonological awareness tasks was at the same level as that of the DS comparison group with similar reading skill, suggesting that bilingualism offers no specific advantage or disadvantage in these metalinguistic tasks.

**Table 5:** English performance on tests of literacy skills for MB (score) at T2, compared with that of typically developing monolingual English children and children with Down Syndrome (DS: means, standard deviations), matched to MB on word reading accuracy

Measure (max score)	MB	TDE (*n* = 15)	Point estimate^1^ (95% CI)	DS (*n* = 6)	Point estimate^2^ (95% CI)
Syllable deletion (10)	8	9.80 (0.41)	0.04 (0.00–0.35)**	8.00 (1.67)	50.00 (21.18–78.82)
Phoneme deletion (10)	3	7.47 (2.03)	2.56 (0.08–10.78)†	2.20 (2.17)	62.33 (28.70–89.60)
Initial phoneme isolation (10)	7	9.33 (1.23)	4.40 (0.31–15.30)†	8.83 (1.47)	15.06 (1.04–45.27)
Final phoneme isolation (10)	9	8.53 (1.85)	59.54 (39.56–77.77)	8.17 (1.72)	66.31 (34.85–90.55)
Letter‐sound knowledge (32)	28	30.07 (1.94)	15.95 (4.50–33.89)	27.17 (3.71)	57.80 (27.48–84.72)
Single word reading (60)	24	27.73 (4.40)	21.28 (7.62–40.41)	23.33 (6.28)	53.74 (24.16–81.71)
Word reading (15)	8	12.33 (2.85)	8.17 (1.19–22.53)	9.00 (3.58)	40.31 (13.92–70.93)
Nonword reading (15)	1	10.20 (3.75)	1.62 (0.03–7.91)*	2.83 (2.40)	25.59 (4.87–57.43)
Nonword reading (20)	7	12.00 (5.30)	18.82 (6.12–37.49)	2.17 (2.64)	92.45 (67.17–99.92)
Prose reading errors (48)	17	8.80 (5.98)	89.72 (74.07–98.09)	16.33 (12.52)	51.88 (22.67–80.28)
Prose reading comprehension (24)	10	17.07 (2.46)	0.73 (0.00–4.49)*	7.17 (2.32)	84.50 (54.14–98.86)

Notes. ^1^Percentage of the English typically developing population aged 7 years estimated to perform below MB's score.

^2^Percentage of the English DS population aged 8–10 years with age‐appropriate word reading estimated to perform below MB's score. CI = confidence interval.

^†^ *p* < .10, ^*^ *p* < .05, ^**^ *p* < .01.

MB's literacy scores at T2 and the average performance of the DS and TDE comparison groups matched for word reading are given in the lower rows of Table 5 (note that MB was being assessed in her L2 while the comparison groups in L1). MB's word‐level reading did not differ significantly from that of the comparison groups (in line with the matching procedure); her performance on the experimental test of nonword reading was significantly below that of the TDE comparison group but it was statistically equivalent on the standardized test. Her reading comprehension was significantly weaker. When compared with the DS comparison group, there was a trend for MB's nonword reading (on the standardized measure) and reading comprehension to be stronger.

### Does MB Show the Same Reading Profile in L1 (Russian) as in L2 (English)?

To assess MB's Russian reading skills, we considered her performance on the Russian reading measures and compared this to scores achieved on parallel English reading tests (first two columns in Table 6). Though MB knew fewer Russian letter sounds than English letter sounds, MB achieved the same score for word and nonword reading across the Russian and English forms of the tests.

**Table 6:** English and Russian performance for MB (score) at T2, compared with that of typically developing monolingual Russian children (means, standard deviations), matched to MB on word reading accuracy

	MB	MB	TDR	Point estimate^1^
Measure (max score)	English	Russian	(*n* = 11)	(95% CI)
Syllable deletion (10)	8	1	2.09 (1.81)	28.85 (10.83–52.23)
Phoneme deletion (10)	3	2	2.09 (1.87)	48.21 (26.17–70.69)
Initial phoneme isolation (10)	7	8	8.45 (1.57)	39.47 (18.84–62.70)
Final phoneme isolation (10)	9	6	6.36 (1.80)	42.60 (21.41–65.62)
Letter‐sounds known (%)	85%	58%	—	—
Letter‐sound knowledge (33)	–	19	28.55 (3.17)	0.81 (0.00–5.78)*
Nonword reading (15)	1	1	7.00 (1.83)	0.61 (0.00–4.88)*
Word reading (15)	4	4	5.00 (1.00)	18.05 (4.31–40.06)

Notes. ^1^Percentage of the Russian typically developing population aged 6 years estimated to perform below MB's score. CI = confidence interval.

^*^ *p* < .05.

Table 6 further contrasts MB's Russian literacy scores with those of the monolingual TDR comparison group. Although Russian is MB's L1, unlike the comparison group, she did not receive formal reading instruction in this language. Relative to the monolingual Russian typically developing group (Table 6), MB's phonological awareness scores were very similar, but her letter‐sound knowledge was weaker. As would be expected given the matching criterion, MB performed similarly to the comparison group on the test of word reading; however, her nonword reading scores were significantly lower.

To summarize our findings, MB has a learning disability as expected for a child with DS, but her verbal skills are relatively well developed; she is a balanced Russian‐English bilingual. MB's performance on literacy tasks was stronger in English than in Russian. It is noteworthy that, consistent with the typical DS reading profile, she had poorer reading comprehension than accuracy in English, and nonword reading was at floor in both languages. However, surprisingly, her phonological awareness was not found to be impaired in Russian.

## Discussion

The current investigation was a case study of MB—a young girl with DS who is bilingual in Russian (L1) and English (L2). Using parallel tasks, we assessed her language and literacy skills in each language. We also evaluated her performance relative to monolingual comparison groups of the same level of word reading. In doing so, we provided the first consideration of both bilingual and biliterate attainments in DS. We focused on comparisons with children matched on word reading ability to determine if MB's profile suggests she learned to read following the typical course or as expected given her language learning impairments.

### MB's Cognitive Profile

MB is functioning at the level of a child with intellectual disability in terms of nonverbal cognitive skills. However, in contrast to what is considered typical for DS, she has well‐developed oral language skills even though her receptive vocabulary is weak. While it would be tempting to conclude that bilingualism has conferred an advantage for MB's language development, the fact that she was performing similarly to monolingual children with DS who were matched for reading level does not support this hypothesis.

### MB's L2 Reading Skills and Reading Progress

MB provides an exceptional example of reading performance in DS. Word‐level reading in her L2—a language to which she was only fully exposed from school entry (age 4)—was consistently at age‐expected levels on standardized tests over a 2.5‐year period (as good as that of typically developing peers reading in their L1). Although word reading is a relative strength in DS, it is uncommon for it to be in line with chronological age (Groen et al., 2006). Indeed, Hulme et al. (2012) reported that this level was only reached by 8% of children with DS who were included in their longitudinal study of L1 literacy development. Consistent with this finding, when compared at T1 to an opportunity sample of 51 children with DS reading in their first and only language (Burgoyne et al., 2012), MB's word reading in her L2 was at a level better than 91% of the sample, despite MB being the youngest child. Thus, this case study also aligns with that of Groen et al. (2006) in demonstrating that competent levels of word reading can be achieved in children with DS. Another similarity was that MB's reading comprehension was not in step with word reading accuracy, but fell in the low‐ to below‐average range. Thus, MB shows a “poor comprehender” reading profile—a typical outcome for children with DS who achieve good levels of word reading (Groen et al., 2006; Nash & Heath, 2011).

### Bilingualism Advantage for L2 Reading

We now turn to consider whether there was any indication that MB's Russian‐English bilingual background had impacted her reading and related skills in either language. Although the findings of a case study cannot be used to attribute causal significance, they can be instructive in highlighting possible associations between bilingualism and reading where there are differences from normal expectation. First, in the comparison with English typically developing readers, we sought evidence as to whether MB showed the typical DS cognitive profile. As expected, she showed low levels of nonverbal ability and vocabulary knowledge relative to the TDE group; however, her verbal memory skills were better developed. This is not the typical DS profile, which suggests that she has better than usual verbal memory abilities. While it is tempting to argue that this is a consequence of bilingualism, the fact that she showed no difference in performance from that of the DS comparison group, who were also good readers, argues against this. When compared with this group, MB showed no advantage on any of the language and general cognitive ability measures.

Turning to MB's reading, despite being equated with the typically developing English comparison group on word reading, her strengths and weaknesses in related tasks were typical for a child with DS; she showed weaknesses in phonological awareness, nonword reading (though differences were not significant on the standardized measure), and reading comprehension. Importantly, although bilingualism is thought to confer an advantage on tasks tapping such skills, there was no evidence of an advantage for phonological awareness tasks or for nonword reading relative to monolingual DS readers of similar levels of word reading. Moreover, the finding that MB performed like the monolingual children with DS on the reading comprehension measure suggests that this weakness was associated with DS and not a consequence of bilingualism, which is often the case. Thus, as for oral language, there was no strong evidence that bilingualism confers any advantage or disadvantage in DS.

### Reading Profiles in L1 and L2

Having seen that MB's oral language was at a similar level in L1 (language of home—Russian) and L2 (language of school—English), we were interested in comparing her reading and related skills across languages. Despite knowing fewer Russian letters, MB could read parallel sets of single words equally well in both languages. When compared with Russian children of similar word reading, MB showed a disadvantage in letter‐sound knowledge (perhaps because she had not been taught these explicitly), and her nonword reading skills were poorly developed, despite showing comparable levels of performance in phonological awareness tasks.

In summary, when evaluating MB's performance in L1 and L2, the benefits of formal instruction in English were seen in her word‐reading skills. However, in neither language did nonword decoding keep pace with word‐level reading skills. Arguably, nonword reading in Russian should be relatively easy given the transparency of the language. The fact that MB's deficit transcends orthographies suggests that it is associated with the language profile of DS and is not orthography specific. On the other hand, MB appears to be relatively better at phonological awareness tasks in Russian (relative to the typically developing comparison group) than in English. This is particularly striking because the phonological awareness tasks in English require operations that result in a real word while those in Russian do not always do so. It seems possible, in this light, that MB can use knowledge of more consistent spelling‐sound correspondences in Russian to bootstrap her otherwise poor performance in such metalinguistic tasks.

Finally, we consider whether MB has a cognitive profile that could either explain her success in learning to read or be related to her bilingual proficiency. Unlike many individuals with DS (e.g., Jarrold, Baddeley, & Phillips, 2002), MB had better memory for verbal information than for visuospatial information. Groen et al. (2006) suggested that the child with DS whom they studied may have become an exceptional reader because her verbal memory skills were strong (as was her phonological awareness). However, this child's parents were well educated and it is probable that she benefitted from an excellent home literacy environment, reflecting favorable gene‐environment correlation. The same could be said of MB, suggesting that bilingualism was not the key factor here. Indeed, the word reading matched monolingual children with DS also had good verbal memory skills. On the other hand, the deficit shown by MB in visuospatial memory was unusual (though it is worth noting that she did not differ significantly from the monolingual children with DS on this measure).

## Limitations and Future Research

This study has a number of limitations. First, we acknowledge that it is difficult to draw definitive conclusions from a single case study, albeit with longitudinal data. Ideally the comparison groups would have been followed over time in order to assess possible differences in growth trajectory for language and reading; future research on bilingualism and biliteracy in children with DS should aim to do this. Second, the study included only a limited language assessment in the two languages. Measures beyond vocabulary to include grammar should be part of future research in order to ascertain whether the grammatical impairments observed in DS transcend languages with different syntactic structures. Also further consideration needs to be given to how best to create parallel tasks across languages. Here we focused on phonological structure because of its relevance for learning to read and controlled for as many psycholinguistic variables as possible, attempting to ensure equivalent familiarity with the words used in the two languages. However, other controls are certainly possible and may affect results.

To summarize, our findings support the main conclusions of Kay‐Raining Bird et al. (2005) regarding the bilingual attainments of children with DS, but go further by documenting biliterate attainment in DS. We have confirmed that strikingly similar levels of competence can be achieved in two different spoken languages in a child with DS and have extended the finding to word‐level literacy. While it should be acknowledged that MB had received reading intervention along with her English counterparts, it is striking that she had mastered learning to read in two languages with differences in not only the predictability and consistency of spelling to sound correspondences, but also in symbol systems. Recognizing the social advantages of being a flexible language user, there seems no reason to suggest that a child with DS should only be taught to read in one language.

## Conclusion

This case study of MB makes a novel contribution to our understanding of language and literacy development in individuals with DS. We have confirmed that competent levels of word reading are achievable by children with DS and have additionally shown that this can be the case in a child's L2. We have provided the first report of bilingual and biliterate attainments in DS, demonstrating reading acquisition in two different orthographies with different alphabets. We have strengthened the evidence that learning two languages in the presence of a learning disability need have no detrimental effect on a child's language development, and have extended this finding to the realm of word‐level literacy. However, given the wide individual differences among children with DS, the findings need replication before firm educational recommendations can be made. Indeed, we acknowledge that it is not possible to disentangle the effects of a positive genetic endowment and a rich language and literacy environment in understanding the achievements in a single case study of DS.

## Supporting information


**Appendix S1**. Overview of Test Protocol.
**Appendix S2**. Syllable Deletion Task in Russian and English.
**Appendix S3**. Phoneme Deletion Task in Russian and English.
**Appendix S4**. Initial and Final Phoneme Isolation Tasks in Russian and English.
**Appendix S5**. Word and Nonword Reading Tasks in Russian and English.
