# Supplementary material for: Bilingualism and Biliteracy in Down Syndrome: Insights From a Case Study
Source: Lang Learn. 2016 Jun 1;66(4):945–71. doi: 10.1111/lang.12179 (PMC5132129; doi:10.1111/lang.12179)
Supplement: Supplementary file 1 — Appendix S1. Overview of Test Protocol. Appendix S2. Syllable Deletion Task in Russian and English. Appendix S3. Phoneme Deletion Task in Russian and English. Appendix S4. Initial and Final Phoneme Isolation Tasks in Russian and English. Appendix S5. Word and Nonword Reading Tasks in Russian and English. [file LANG-66-945-s001.pdf]

Supporting Information for: Burgoyne, K., Duff, F. J., Nielsen, D., Ulicheva, A., & Snowling, M. J. Bilingualism and biliteracy in Down syndrome: Insights from a case study. Article accepted in *Language Learning* on 12 January 2016.

Appendix S1: Overview of Test Protocol

	Participants			
Tests	MB	DS	TDE	TDR
T1				
Literacy Skills				
YARC Early Word Reading	×			
YARC Single Word Reading	×			
T2				
Nonverbal Ability				
WPPSI-III Block Design and Object Assembly	×	×	×	
WMTBC Block recall	×	×	×	
Verbal Memory				
WMBTC Digit Recall and Word Recall	×	×	×	
Language Skills				
BPVS	×	×	×	
BPVS Russian translation	×			
CELF-IV Expressive Vocabulary	×	×	×	
CELF-IV Expressive Vocabulary Russian translation	×			
Literacy Skills				
Syllable deletion English	×	×	×	
Syllable deletion Russian	×			×
Phoneme deletion English	×	×	×	

Phoneme deletion Russian	×			×
Phoneme isolation English	×	×	×	
Phoneme isolation Russian	×			×
Letter Knowledge English	×	×	×	
Letter Knowledge Russian	×			×
Bespoke Word Reading English	×	×	×	
Bespoke Word Reading Russian	×			×
YARC Early Word Reading	×	×	×	
YARC Single Word Reading	×	×	×	
Bespoke Nonword Reading English	×	×	×	
Bespoke Nonword Reading Russian	×			×
GNWRT Nonword Reading	×	×	×	
YARC passage reading	×	×	×	
T3				
Literacy Skills				
YARC single word reading	×			
YARC passage reading	×			

Appendix S2: Syllable Deletion Task in Russian and English

	Russian			English		
	Test item	Syllable to delete	Answer	Test item	Syllable to delete	Answer
1	цветок /t͡svʲəʻtok/ "flower"	/t͡svʲe/	/tok/ "current"	snowman	snow	man
2	носок /nɐ'cok/ "sock"	/na/ "but"	/sok/ "juice"	seaside	sea	side
3	больной /bɐlʲ'noj/ "patient"	/noj/	/bolʲ/ "pain" OR /bɐlʲ/	bedroom	room	bed
4	ножка /'noʂkə/ "leg"	/ka/	/noʂ/ "knife"	rainbow	bow	rain
5	забор /zɐ'bor/ "fence"	/za/ "behind"	/bor/ "forest"	teapot	tea	pot
6	чайник /'t͡ɕajnʲək/ "teapot"	/nʲik/	/t͡ɕaj/ "tea"	football	ball	foot

7	дворник /’dvornʲək/ “yard-keeper”	/nʲik/	/dvor/ “yard”	toothbrush	brush	tooth
8	пирог /pʲə’rok/ “pie”	/pʲi/	/rok/ “horn”	rowboat	row	boat
9	город /’gorət/ “city”	/go/	/rot/ “mouth” OR /rat/ “glad”	peanut	pea	nut
10	мечта /mʲət͡ɕ’ta/ “dream”	/ta/	/mʲet͡ɕ/ “sword” OR /mʲit͡ɕ/	jigsaw	saw	jig

Note. If translation is not given, then the sequence of phonemes does not represent an existing word in Russian.

Appendix S3: Phoneme Deletion Task in Russian and English

	Russian			English		
	Test item	Phoneme to delete	Answer	Test item	Phoneme to delete	Answer
1	танк /tank/ “tank”	/n/	/tak/ “so”	nest	s	net
2	смех /smʲex/ “laugh”	/s/	/mʲex/ “fur”	smile	s	mile
3	ручка /'rut͡ɕkə/ “pen”	/t͡ɕ/	/rə'ka/ “hand”	window	n	widow
4	зуб /zup/ “tooth”	/p/	/zu/	team	m	tea
5	нож /noʂ/ “knife”	/n/	/oʂ/	ball	b	all
6	рот /rot/ “mouth”	/r/	/ot/ “from”	meal	m	eel
7	двор /dvor/ “yard”	/d/	/vor/ “thief”	cloud	c	loud
8	Нос /nos/ “nose”	/s/	/no/ “but”	bike	k	by
9	Врун /vrun/ “liar”	/n/	/vru/ “I lie”	train	n	tray
10	Сок /sok/ “juice”	/k/	/so/	pile	l	pie

Note. Order of item presentation for English items was: ball, meal, cloud, smile, team, bike, train, pile, window, nest.

Appendix S4: Initial and Final Phoneme Isolation Tasks in Russian and English

	Initial phoneme isolation				Final phoneme isolation			
	Russian		English		Russian		English	
	Test item	Answer	Test item	Answer	Test item	Answer	Test item	Answer
1	Кот /kot/ “cat”	/k/	cat	k	Нож /noʂ/ “knife”	/ʂ/	mug	g
2	Брюки /brʲukʲə/ “trousers”	/b/	spider	s	Стол /stol/ “table”	/l/	bread	d
3	Мяч /mʲat͡ɕ/ “ball”	/m/	leg	l	Утюг /u’tʲuk/	/k/	oven	n
4	цветок /t͡svʲə‘tok/ “flower”	/t͡s/	planet	p	Арбуз /ɐr’bus/ “watermelon”	/s/	atlas	s
5	Стул /stul/ “chair”	/s/	clock	k	Банан /bɐ’nan/ “banana”	/n/	rabbit	t
6	Тарелка /tə’rʲelkə/ “plate”	/t/	factory	f	Перец /’pʲerʲət͡s/ “pepper”	/t͡s/	hammock	k
7	Лампа /’lampə/ “lamp”	/l/	window	w	Шкаф /ʂkaf/ “wardrobe”	/f/	pram	m
8	Кровать /krɐ’vatʲ/ “bed”	/k/	dragon	d	цветок /t͡svʲə‘tok/ “flower”	/k/	cobweb	b
9	Зонт /zont/ “umbrella”	/z/	tent	t	Бант /bant/ “bow”	/t/	lamp	p

10	Хлеб /xlʲep/ “bread”	/x/	frog	f	Сыр /sɨr/ “cheese”	/r/	dish	sh

Appendix S5: Word and Nonword Reading Tasks in Russian and English

	Words		Nonwords	
	Russian	English	Russian	English
1	Слон /slon/ “elephant”	tram	Дуг /duk/	fet
2	Сад /sat/ “garden”	bed	Туль /tulʲ/	joth
3	Мост /most/ “bridge”	dust	Луп /lup/	vas
4	Гриб /grʲip/ “mushroom”	crab	Шень /ʂenʲ/	weth
5	Ёлка /'jolkə/ “pine tree”	twin	Коле /kolʲe/	reco
6	Торт /tort/ “cake”	sand	Расть /rastʲ/	culch
7	Гвоздь /'gvostʲ/ “nail”	plant	Кмас /kmas/	brep
8	Взгляд /vzglʲat/ “look”	napkin	Моза /moza/	kety
9	Школа /'ʂkolə/ “school”	stamp	Напа /napa/	loda
10	Скрипка /'skrʲipkə/ “violin”	pumpkin	Ланка /lanka/	lesta
11	Детство /'det͡stvə/ “childhood”	nostril	Несто /nʲesto/	birgo
12	Равнина /rɐv'nʲinə/ “plain”	plastic	Полос /polos/	nivel
13	Поддержка /pɐ'ddʲerʂkə/ “support”	bandstand	Пасок /pasok/	halon
14	Животное /ʐə’votnəjə/ “animal”	skeleton	Снечка /snʲet͡skə/	prento
15	Велосипед /vʲəlɐsʲə’pʲet/ “bicycle”	tarantula	Порехи /porʲexʲi/	tenama

Note. For nonwords, any stress pattern was considered correct. In Russian the pronunciation of vowels depends on stress assignment (unstressed vowels become reduced). Therefore, no vowel reduction is marked in the transcription.
